# Supplementary material for: Genetic vitamin B6 deficiency and alcohol interaction in behavior and metabolism
Source: bioRxiv. 2025 Mar 7:2025.03.06.641947. Preprint. [Version 1] doi: 10.1101/2025.03.06.641947 (PMC11908246; doi:10.1101/2025.03.06.641947)
Supplement: Supplement 7 [file NIHPP2025.03.06.641947v1-supplement-7.pdf]

bioRxiv preprint doi: https://doi.org/10.1101/2025.03.06.641947; this version posted March 7, 2025. The copyright holder for this preprint (which was not certified by peer review) is the author/funder, who has granted bioRxiv a license to display the preprint in perpetuity. It is made available under aCC-BY 4.0 International license.

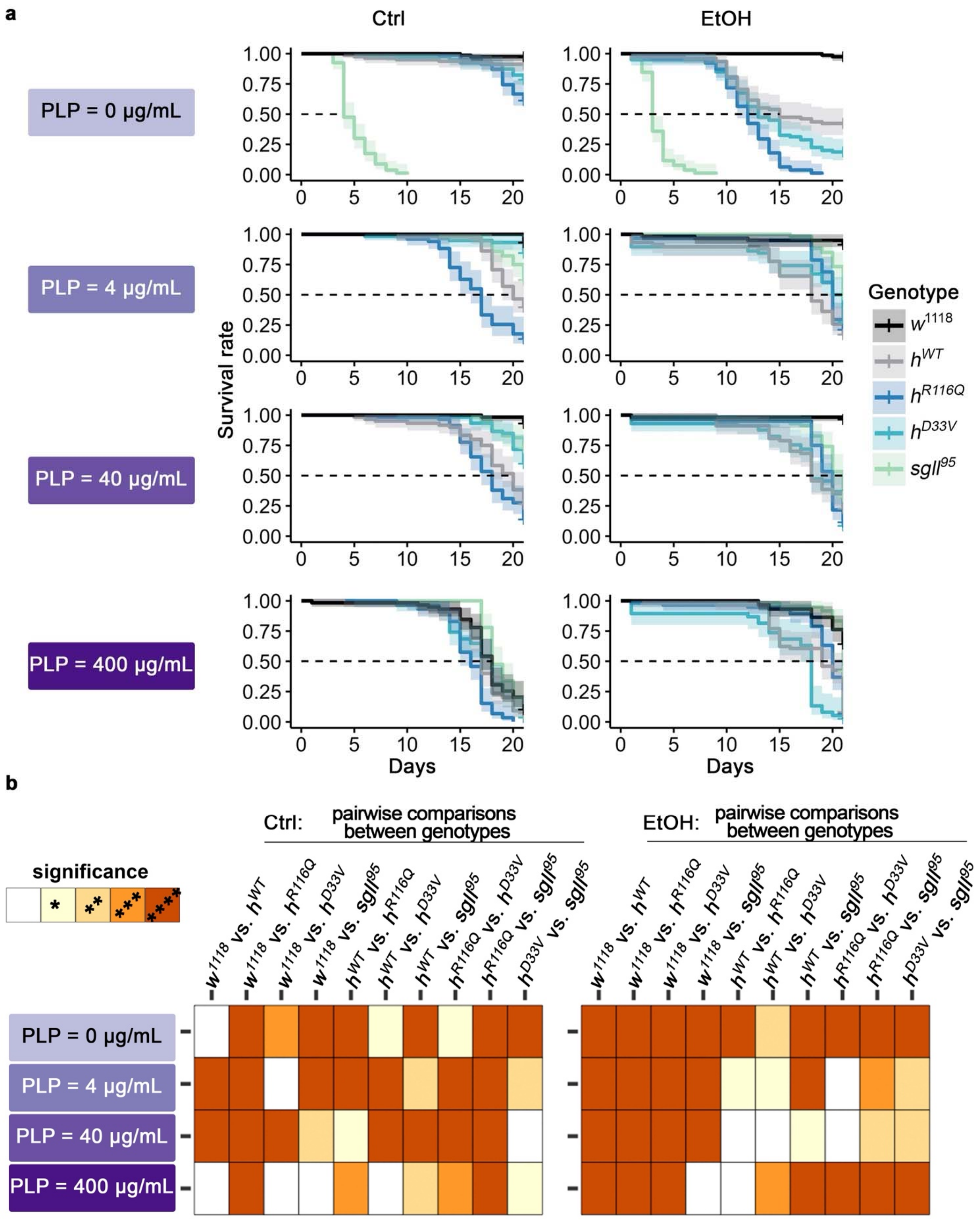

Supple. Fig. 1: Effects of chronic alcohol exposure and PLP supplementation on survival of flies from each genotype. a Survival of flies from each genotype on control diet (Ctrl) or 16% alcohol (EtOH) for 21 days in combination with PLP supplementation. *n*=38-80 flies per genotype per diet treatment. Log-rank test with Benjamini-Hochberg corrections. **b** Pairwise log-rank test with Benjamini-Hochberg corrections. *p<0.05,

bioRxiv preprint doi: https://doi.org/10.1101/2025.03.06.641947; this version posted March 7, 2025. The copyright holder for this preprint (which was not certified by peer review) is the author/funder, who has granted bioRxiv a license to display the preprint in perpetuity. It is made available under aCC-BY 4.0 International license.

**p<0.01, ***p<0.001, ****p<0.0001. See Supplementary Tables 1-2 for exact n in each condition and exact p values for comparisons.

bioRxiv preprint doi: https://doi.org/10.1101/2025.03.06.641947; this version posted March 7, 2025. The copyright holder for this preprint (which was not certified by peer review) is the author/funder, who has granted bioRxiv a license to display the preprint in perpetuity. It is made available under aCC-BY 4.0 International license.

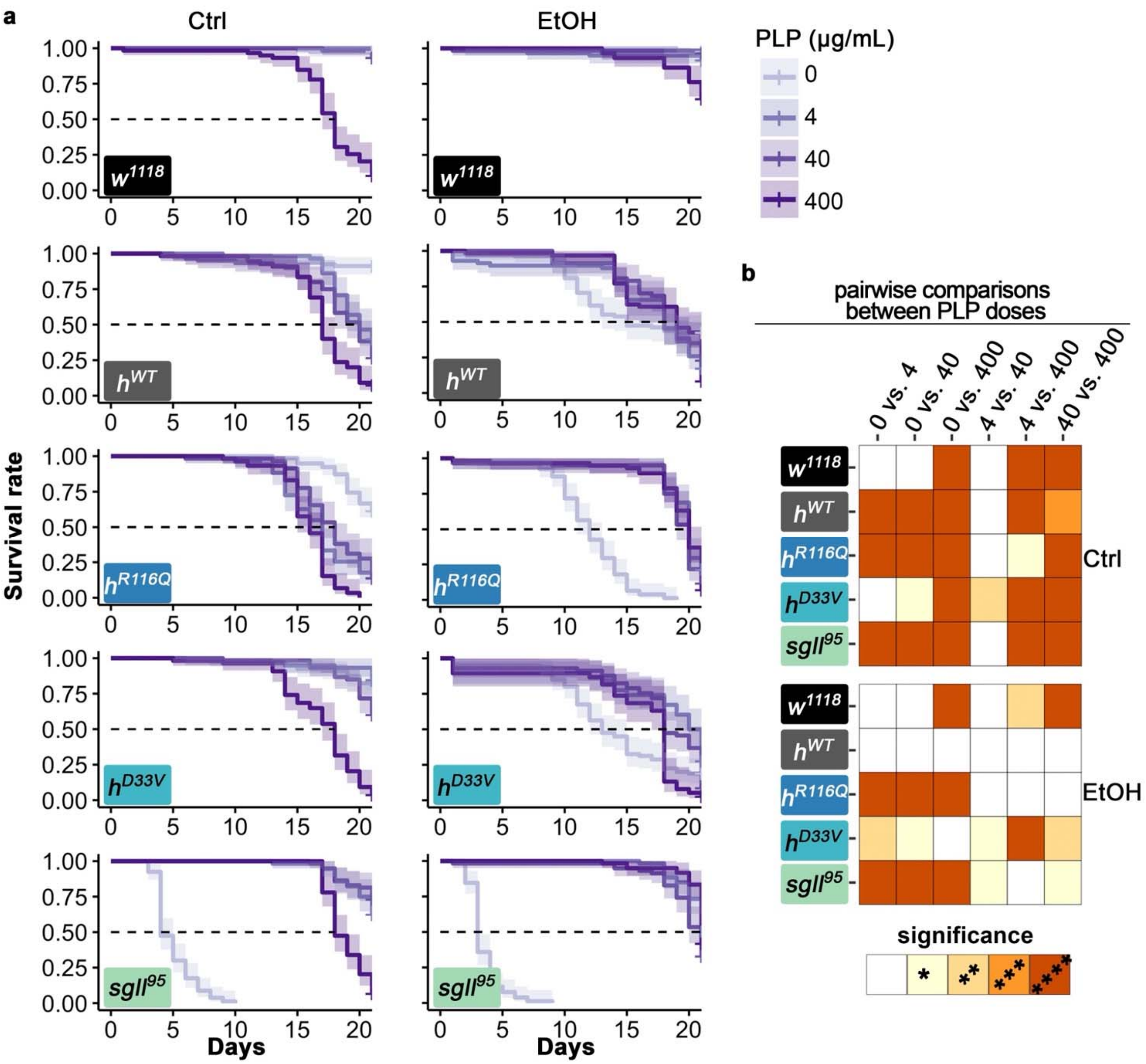

Supple. Fig. 2: Effects of chronic alcohol exposure and PLP supplementation on survival of flies from each genotype. a Survival of flies from each genotype on control diet (Ctrl) or 16% alcohol (EtOH) for 21 days in combination with PLP supplementation. *n*=38-80 flies per genotype per diet treatment. **b** Pairwise log-rank test with Benjamini-Hochberg corrections. *p<0.05, **p<0.01, ***p<0.001, ****p<0.0001. See Supplementary Tables 1-2 for exact n in each condition and exact p values for comparisons.

bioRxiv preprint doi: https://doi.org/10.1101/2025.03.06.641947; this version posted March 7, 2025. The copyright holder for this preprint (which was not certified by peer review) is the author/funder, who has granted bioRxiv a license to display the preprint in perpetuity. It is made available under aCC-BY 4.0 International license.

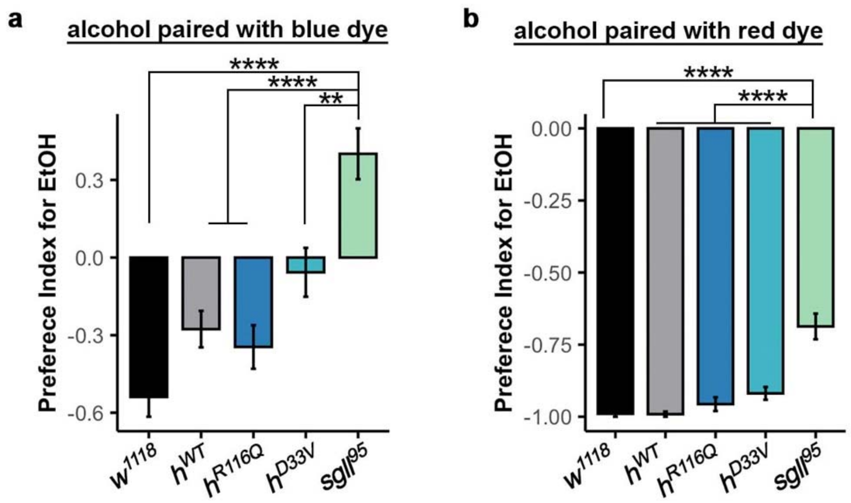

Supple. Fig. 3: Preference Index for EtOH over isocaloric maltodextrin. *n*=8 and 9 assays per genotype for alcohol paired with blue dye and paired with red dye, respectively. Data is presented as mean ± SEM. One-way ANOVA with Tukey's post hoc. n.s.:p>0.05, *p<0.05, **p<0.01, ***p<0.001, ****p<0.0001. See Supplementary Table 2 for exact p values.

bioRxiv preprint doi: https://doi.org/10.1101/2025.03.06.641947; this version posted March 7, 2025. The copyright holder for this preprint (which was not certified by peer review) is the author/funder, who has granted bioRxiv a license to display the preprint in perpetuity. It is made available under aCC-BY 4.0 International license.

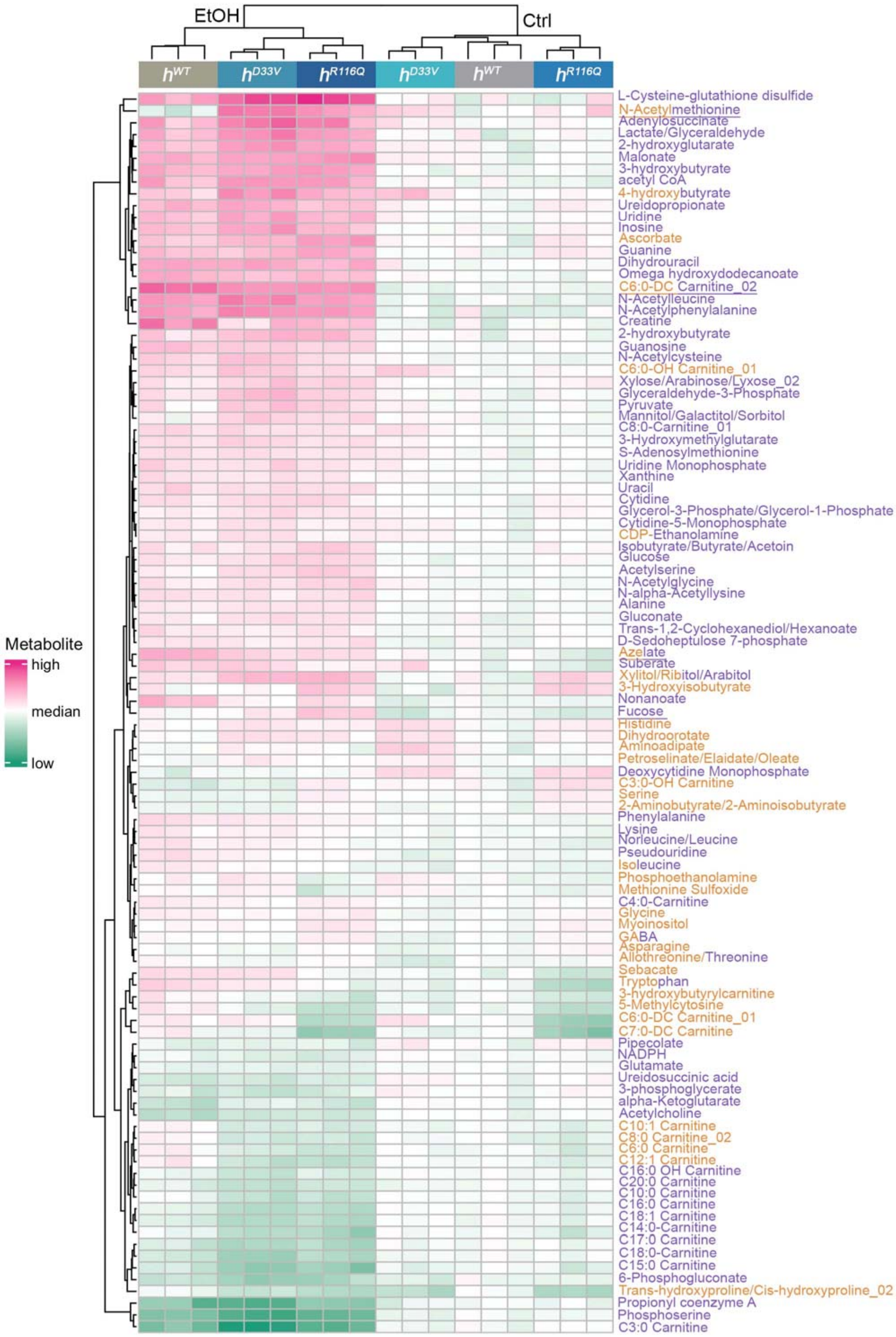

bioRxiv preprint doi: https://doi.org/10.1101/2025.03.06.641947; this version posted March 7, 2025. The copyright holder for this preprint (which was not certified by peer review) is the author/funder, who has granted bioRxiv a license to display the preprint in perpetuity. It is made available under aCC-BY 4.0 International license.

Supple. Fig. 4. **Hierarchical clustering of metabolites.** Only metabolites with FDR<0.05 are included. Hierarchical clustering on both samples (columns) and metabolites (rows). Metabolites are color-coded. Orange and purple indicate significant metabolites for genotype and treatment, respectively. Metabolites with dual colors are significant for both genotype and treatment. Metabolites with underlines are significant for genotype-by-treatment interaction.
